# Analysis of choroidal thickness in patients with proliferative diabetic retinopathy by optical coherence tomography angiography

**DOI:** 10.12669/pjms.37.7.4357

**Published:** 2021

**Authors:** Qian Ren, Hua Yu, Zhaohui Sun, Li Li

**Affiliations:** 1 Qian Ren, Department of Ophthalmology, Shijiazhuang People’s Hospital, Shijiazhuang, 050011, Hebei, China; 2 Hua Yu, Department of Ophthalmology, Shijiazhuang People’s Hospital, Shijiazhuang, 050011, Hebei, China; 3 Zhaohui Sun, Department of Ophthalmology, Shijiazhuang People’s Hospital, Shijiazhuang, 050011, Hebei, China; 4 Li Li, Department of Ophthalmology, Shijiazhuang People’s Hospital, Shijiazhuang, 050011, Hebei, China

**Keywords:** Diabetic retinopathy, Proliferative, Optical coherence tomography angiography

## Abstract

**Objectives:**

This study aimed to evaluate the changes in choroidal thickness at different parts in patients with stage IV-V diabetic retinopathy (DR) treated by Panretinal Photocoagulation (PRP) or its combination with anti-vascular endothelial growth factor (VEGF) therapy using optical coherence tomography angiography (OCTA).

**Methods:**

Patients with proliferative DR (stage IV-V) diagnosed in Shijiazhuang People’s Hospital between January 2016 to January 2020 were selected and treated with conventional PRP or combined with anti-VEGF therapy. OCTA was performed before treatment and one three and six months after treatment to observe and compare subfoveal choroidal thickness (SFCT), perifoveal choroidal thickness at 500 um (M500) and 1500 um (M1500).

**Results:**

A total of 76 patients (133 eyes) were included. Six months later, re-examination showed effective treatment in 122 eyes (91.72%) and ineffective treatment in 11 eyes. Before treatment and one week, three months and six months after treatment, the choroidal thickness was observed and compared by OCTA. SFCT, M500 and M1500 increased one week after treatment, were significantly thinner 3 months after treatment than those before treatment, and further decreased six months after treatment.

**Conclusion:**

OCTA presents a good evaluation of perifoveal choroidal thickness in patients with proliferative DR. It provides a basis for treatment selection and efficacy determination of proliferative DR.

## INTRODUCTION

Diabetic retinopathy (DR) is the most common complication of diabetes in the late stage.^1^-^3^ It can involve retinal and choroidal capillaries, central retinal artery, ciliary artery and ophthalmic artery, resulting in severely decreased vision.^1^-^3^ At present, the clinical diagnosis and staging of DR are mainly based on ophthalmoscopic examination, Doppler ultrasound, indocyanine green angiography (ICGA), fluorescein angiography (FA) and optical coherence tomography (OCT).^4^,^5^ However, these methods have their limitations in clinical application. Recently, optical coherence tomography angiography (OCTA) has been widely used in clinical practice.^4^-^10^ OCTA can be used for non-invasive, high-speed, high-resolution and multi-level three-dimensional angiography and to further quantitatively analyses retinal vascular density, non-perfusion area and blood flow indexes with relevant software. Therefore, it can display retinal structure and analyses retinal function.^4^-^10^ Currently, there is no uniform treatment for proliferative DR in stage IV-V at home and abroad. The patients are routinely treated with Panretinal photocoagulation (PRP) or its combination with anti-vascular endothelial growth factor (VEGF) therapy. Even after several additional laser treatments, some patients eventually have to undergo pars plana vitrectomy, and their visual acuity is not significantly improved.^11^-^16^ Therefore, the patients should be evaluated through examinations to choose the treatment timing and method scientifically and rationally. In this study, the efficacy of PRP or its combination with anti-VEGF therapy in the treatment of stage IV-V DR was evaluated using OCTA, providing a basis for the diagnosis and treatment of proliferative DR.

## METHODS

### Clinical data

From January 2016 to January 2020, 76 patients (133 eyes) who met the diagnostic criteria of stage IV-V DR were selected. There were 41 males (76 eyes) and 35 females (57 eyes), with an average age of 52.10 ± 9.27 years and a course of diabetes of 11.54 ± 4.27 years. Additionally, DR was found in both eyes in 57 patients (114 eyes) and in one eye in 19 patients (19 eyes). All the included patients routinely received visual examination, slit-lamp examination, indirect ophthalmoscopy, intraocular pressure testing and B-Scan Ultrasound examination of the eyes. Subfoveal choroidal thickness (SFCT), perifoveal choroidal thickness at 500 um (M500) and 1500 um (M1500) were evaluated using OCTA.

### Ethical approval

The study was approved by the Institutional Ethics Committee of Shijiazhuang People’s Hospital and written informed consent was obtained from all participants.

### Inclusion criteria

(1) Patients met the diagnostic criteria of stage IV-V DR. (2) Patients had no other ocular lesions and no history of laser treatment or intraocular surgery. (3) Underlying diseases were controlled, fasting blood glucose was lower than 8 mmol/L and liver and kidney function indices were not higher than 1.5 times. (4) The refractive medium was clear and did not affect OCTA and PRP or its combination with anti-VEGF therapy.

All the patients were scanned for choroidal thickness using optical coherence tomography (SPECTRALIS OCT; Heidelberg, Germany). Each patient was scanned three times and the average was obtained. According to the examination results, the patients were given PRP, and anti-VEGF therapy was combined if necessary. One week, 3 months and 6 months after treatment, OCTA was performed. The best-corrected visual acuity was recorded before treatment and at least half a year after treatment, and SFCT, M500 and M1500 were observed and compared. Visual acuity examination, slit-lamp examination, indirect ophthalmoscopy, intraocular pressure testing and B-Scan Ultrasound examination of the eyes were conducted at each follow-up. The changes in visual acuity and choroidal thickness were compared to evaluate the efficacy.

### Efficacy evaluation

Efficacy was determined using the quasi-logarithmic visual acuity chart. After treatment, significant improvement in visual acuity (i.e., logarithmic visual acuity increased by 0.2 or more) was considered as significant effective, no change or slight improvement in visual acuity (i.e., logarithmic visual acuity increased by 0.1 or was unchanged) as effective and decrease in visual acuity as invalid.

### Statistical analysis

Statistical analysis was performed using SPSS19.0. The enumeration data were expressed as rate and compared between groups by the χ2 test with row × line data. The measurement data were expressed as x ± s and analyzed using the paired t-test. *P* <0.05 was considered statistically significant.

## RESULTS

One week, 3 months and 6 months after PRP combined with anti-VEGF therapy, the efficacy was determined according to the standard logarithmic visual acuity chart. The visual acuity improved or remained unchanged in 92 eyes one week after treatment, with an overall effective rate of 73.6%. Three months after treatment, visual acuity improved or unchanged in 106 eyes, with an overall effective rate of 84.8%. Six months after treatment, 115 eyes showed improved or unchanged visual acuity, with an overall effective rate of 92% (Table-I).

**Table I:** Improved visual acuity at different stages after treatment (%).

*Group*	*After 1 week*	*After 3 months*	*After 6 months*
Improved or unchanged visual acuity	97	113	122
Decreased visual acuity	36	20	11
Effective rate (%)	72.93	84.96	91.72

SFCT, M500 and M1500 were measured before and after treatment. It was observed that one week after treatment, SFCT, M500 and M1500 increased in various degrees, which were statistically significant compared with those before treatment (*P* <0.05). It was considered to be related to retinal oedema at the beginning of treatment. Three months after treatment, the thickness was significantly thinner than those before treatment (*P* <0.05) and further decreased 6 months after treatment (*P* <0.05) (Table-II).

**Table II:** Changes of choroidal thickness in different parts before and after treatment (um, X ± s).

*Group*	*Number of eyes*	*Before treatment*	*One week after treatment*	*Three months after treatment*	*Six months after treatment*
SFCT	133	336.17 ± 12.46	358.81 ± 15.36*	325.64 ± 10.20**	314.91 ± 11.88***
M500	133	302.84 ± 22.91	324.29 ± 13.36*	293.76 ± 23.09**	284.32 ± 15.35***
M1500	133	285.25 ± 12.67	293.03 ± 10.79*	277.35 ± 12.82**	271.59 ± 14.72***

* Compared with before treatment, P < .05,

** Compared with before treatment, P < .05,

*** Compared with before treatment, P < .05.

## DISCUSSION

Diabetic retinopathy (DR) is always a common ocular complication of diabetic patients due to abnormal glucose metabolism. It is the leading cause of blindness in developed countries such as Europe and the United States. In recent years, DR in China has been increasing annually.^1^-^3^ ICGA and FA are widely used in the Clinical practice, as they can evaluate the microvascular circulation of the retina and choroid. However, these two examinations are invasive and time-consuming.

However, once fluorescein leakage occurs, the transparency of the refractive medium will be poor, thereby influencing chorioretinal two-dimensional imaging.^4^ OCT is convenient, non-invasive, non-contact and repeatable technique and can display the fine structure of each layer of the retina.^4^ With the continuous development of OCT software and hardware, imaging technology OCTA, which is widely used in clinical practice, has further improved OCT from structural imaging to functional imaging of local structures.^4^-^10^ The imaging principle of OCTA can be summarized as follows:

The same part of the fundus is scanned rapidly, with the same time interval. The stable signals generated by static tissues and the signals of moving tissues generated by moving particles in backscattered light signals are analyzed. The stable signals generated by static tissues will not change, whereas the signals of moving tissues will change in real time. Therefore, after filtering static signals, the remaining moving signals are the intravascular blood flow information of the fundus. Finally, the retina and choroid angiography is achieved through a series of processing.^4^-^10^ In the current research, many scholars have confirmed that OCTA has a high resolution, operability and repeatability in imaging retinal vessels at different levels.^4^-^10^ OCTA has more advantages than FA in the image clarity and hierarchical observation of retinal non-perfusion area and new blood vessels.^4^-^10^ At present, OCTA is used clinically to observe the number of microaneurysms in the retina at different layers and the retina’s non-perfusion area in DR. Moreover, OCTA can evaluate the retinal vascular density in patients with DR at various stages.^4^,^5^ For those patients injected with anti-VEGF drugs, OCTA can be used to evaluate the changes in the fundus structure and blood vessels before and after injection to formulate individualized treatment for different patients according to the OCTA results.^13^

At present, DR cannot be treated effectively in the clinic. It can only be delayed, so it remains the leading disease that causes blindness.^1^ Our commonly used treatment methods include anti-VEGF therapy, laser treatment, vitrectomy and others.^11^-^16^ Clinically, intravitreal injection of anti-VEGF drugs is mainly used to treat diabetic macular oedema (DME) and proliferative DR.^11^,^13^ Studies have shown that the VEGF level in the vitreum and serum of diabetic patients is significantly higher than normal individuals. VEGF is closely related to the occurrence and development of DR and DME.^11^-^14^ VEGF expression in the vitreous cavity of DME patients increases significantly, further damaging the blood-retinal barrier and increasing the retinal permeability, resulting in DME.^11^,^13^ Anti-VEGF drugs can penetrate the choroid, thus counteracting VEGF’s effect in the large choroidal vessels and choroidal capillaries and further improving patients’ visual acuity by reducing angiogenesis.^11^-^14^ Clinically, macular oedema treatment involving the fovea is difficult. Intravitreal injection of anti-VEGF drugs can be performed based on macular laser photocoagulation and vitreous hormone injection.^11^-^16^ Current studies have shown that anti-VEGF drugs combined with laser photocoagulation in macular oedema treatment of DR can reduce choroidal neovascularisation and choroidal thickness to maintain the current visual acuity or improve the visual acuity of patients.^13^,^14^ Xu Jianlong et al.^16^ observed the efficacy of modified PRP in the treatment of DR and its effect on patients’ VEGF level, and revealed that modified PRP is safe and effective in the treatment of DR. In addition, PRP could promote the regression of retinal non-perfusion area and neovascularisation, and further reduce the VEGF level, thus improving patients’ visual acuity by improving retinal ischaemia and hypoxia.

PRP is currently recognised as a safe and effective measure for the treatment of DR worldwide.^14^-^16^ PRP is effective in the treatment of pre-proliferative DR without needing additional photocoagulation or vitrectomy. Proliferative DR at the early stages generally requires multiple additional photocoagulations, and most patients can obtain stable visual acuity. As for high-risk proliferative DR, the patients’ condition cannot always be controlled after multiple additional photocoagulations, and vitrectomy may be needed.^14^-^16^ PRP’s effect on choroidal thickness varies greatly with the time after treatment.^14^-^16^ It has been shown that the choroidal thickness increases in the first week after PRP but decreases significantly in DR patients 4 weeks, 12 weeks and 12 months after PRP. The thickening of choroidal thickness may be due to the increase in choroidal vascular permeability caused by PRP, leading to choroidal oedema and thickening. In addition, laser therapy may induce choroidal injury, resulting in neovascularisation or redistribution at spots. Therefore, there is a theoretical basis for anti-VEGF drug application after PRP.^17^,^18^ Further, some scholars have found that three months after focal photocoagulation, DR patients’ choroidal thickness does not change significantly and is not related to photocoagulation frequency.

The results of our study are in line with the previous studies. Six months later, re-examination revealed that treatment was effective in 122 eyes (91.72%) and ineffective in 11 eyes (8.37%). Before treatment and one week, three months and six months after treatment, the choroidal thickness was observed and compared by OCTA. SFCT, M500 and M1500 increased one week after treatment and were significantly thinner than those before treatment at three months and further decreased at six months.

### Limitations of this study

The number of subjects included in this study is limited, so the conclusions drawn may not be very convincing. In addition, we only analyzed the cases included in our hospital, which may not be representative enough. We look forward to a multi-center study in the future to reach more comprehensive conclusions.

## CONCLUSION

OCTA has special importance in evaluating perifoveal choroidal thickness in proliferative DR patients. It provides a basis for treatment selection and efficacy determination of proliferative DR.

### Authors’ Contributions:

**QR &** **LL:** Designed this study and prepared this manuscript and are responsible and accountable for the accuracy or integrity of the work.

**HY:** Collected and analysed clinical data.

**ZS:** Significantly revised this manuscript.

## References

[1] Stitt AW, Curtis TM, Chen M, Medina RJ, McKay GJ, Jenkins A (2016). The progress in understanding and treatment of diabetic retinopathy. Prog Retin Eye Res.

[2] Chua J, Sim R, Tan B, Wong D, Yao X, Liu X (2020). Optical Coherence Tomography Angiography in Diabetes and Diabetic Retinopathy. J Clin Med.

[3] Moran EP, Wang Z, Chen J, Sapieha P, Smith LE, Ma JX (2016). Neurovascular cross talk in diabetic retinopathy:Pathophysiological roles and therapeutic implications. Am J Physiol Heart Circ Physiol.

[4] Tey KY, Teo K, Tan ACS, Devarajan K, Tan B, Tan J (2019). Optical coherence tomography angiography in diabetic retinopathy:a review of current applications. Eye Vis (Lond).

[5] Muye L, Xuedong Z (2020). Research advances on clinical application of OCTA in diabetic retinopathy. Recent Adv Ophthalmol.

[6] Parodi MB, Iacono P, Ravalico G, Bandello F (2011). Subthreshold laser treatment for retinal arterial macroaneurysm. Br J Ophthalmol.

[7] Rommel F, Siegfried F, Kurz M, Brinkmann MP, Rothe M, Rudolf M (2018). Impact of correct anatomical slab segmentation on foveal avascular zone measurements by optical coherence tomography angiography in healthy adults. J Curr Ophthalmol.

[8] Perrott-Reynolds R, Cann R, Cronbach N, Neo YN, Ho V, McNally O (2019). The diagnostic accuracy of OCT angiography in naive and treated neovascular age-related macular degeneration:a review. Eye (Lond).

[9] Xiaohui R, Ziqing G (2020). The changes of retinal thickness and blood flow in macular area after anti-VEGF therapy in diabetic macular edema were observed by OCTA. J Clin Ophthalmol.

[10] Weng S, Mao L, Yu S, Gong Y, Cheng L, Chen X (2016). Detection of Choroidal Neovascularization in Central Serous Chorioretinopathy Using Optical Coherence Tomographic Angiography. Ophthalmologica.

[11] Dai Y, Zhou H, Chu Z, Zhang Q, Chao JR, Rezaei KA (2020). Microvascular Changes in the Choriocapillaris of Diabetic Patients Without Retinopathy Investigated by Swept-Source OCT Angiography. Invest Ophthalmol Vis Sci.

[12] Vujosevic S, Toma C, Villani E, Gatti V, Brambilla M, Muraca A (2019). Early Detection of Microvascular Changes in Patients with Diabetes Mellitus without and with Diabetic Retinopathy:Comparison between Different Swept-Source OCT-A Instruments. J Diabetes Res.

[13] Mansour SE, Browning DJ, Wong K, Flynn HW, Bhavsar AR (2020). The Evolving Treatment of Diabetic Retinopathy. Clin Ophthalmol.

[14] Sun JK, Glassman AR, Beaulieu WT, Stockdale CR, Bressler NM, Flaxel C (2019). Rationale and Application of the Protocol S Anti-Vascular Endothelial Growth Factor Algorithm for Proliferative Diabetic Retinopathy. Ophthalmology.

[15] Yibin L (2019). Treatment strategy and development direction of diabetic retinopathy in anti-VEGF era. Ophthalmology in China.

[16] Jianlong X, Qing M (2020). Clinical efficacy of modified panretinal laser photocoagulation in treatment of diabetic retinopathy and its effect on serum VEGF level. Imaging Sci Photochem.

[17] Shuangshuang B, Jihua P, Xin D (2020). Research progress of retinal laser photocoagulation in treatment of diabetic retinopathy. J of Heze Med College.

[18] Juanjuan H, Hongyi S (2020). Clinical evaluation of panretinal photocoagulation in treatment of diabetic retinopathy. Diabetes World.

